# CNT-PDMS foams as self-powered humidity sensors based on triboelectric nanogenerators driven by finger tapping

**DOI:** 10.1038/s41598-023-27690-5

**Published:** 2023-01-07

**Authors:** Mohaddeseh Vafaiee, Faezeh Ejehi, Raheleh Mohammadpour

**Affiliations:** Institute for Nanoscience and Nanotechnology, Sharif University of Technology, Tehran, 14588-89694 Iran

**Keywords:** Sensors and biosensors, Devices for energy harvesting

## Abstract

An increasing number of frequently applied portable electronics has raised the significance of self-powered systems. In this regard, triboelectric nanogenerators (TENGs) have drawn considerable attention due to their diversity of design and high power output. As a widely used material in TENG electrodes, polydimethylsiloxane (PDMS) shows attractive characteristics, such as electron affinity, flexibility, and facile fabrication. To achieve active TENG-based humidity sensing, we proposed a straightforward method to enhance the hydrophilicity of PDMS by two parallel approaches: 1. Porosity induction, 2. Carbon nanotube (CNT) compositing. Both of the mentioned processes have been performed by water addition during the synthesis procedure, which is not only totally safe (in contrast with the similar foaming/compositing routes), but also applicable for a wide range of nanomaterials. Applying the modified electrode as a single-electrode TENG-based humidity sensor, demonstrated an impressive enhancement of sensing response from 56% up to 108%, compared to the bare electrodes. Moreover, the detecting range of ambient humidity was broadened to higher values of 80% in a linear behavior. The fabricated humidity sensor based on a CNT-PDMS foam not only provides superior sensing characteristics but also is satisfactory for portable applications, due to being lightweight and desirably self-powered.

## Introduction

Providing the required energy for launching a large number of sensors depends on supporting the sensing network via numerous power supplies, resulting in frequent battery charging and displacement. A novel approach considered in the past decade is applying the self-powered sensors^1–4^. Since 2012, an increasing number of self-powered systems based on triboelectric nanogenerators (TENG) have been introduced, utilizing mechanical triggers to generate electricity^5–14^. In order to launch a TENG-based sensor, two approaches of passive and active TENG circuits are generally adopted^15^. In passive mode, the resistance of the sensing electrode, which is located outside of the TENG structure, varies by detecting the desired species. By integrating the sensing material as one of the TENG electrodes, the change of the surface charge acts as the detecting mechanism, which is called active TENG-based sensing^16^.

Among the widespread materials applied as TENG electrodes, PDMS (polydimethylsiloxane) is well known for its high electron affinity, as well as straightforward fabrication^17^. Moreover, good mechanical properties, such as high flexibility and tensile strength, provide PDMS as an appropriate candidate for highly repetitive tapping or rubbing cycles in TENGs^18–20^. There is also a wide range of proposed applications for PDMS, due to its inherent biocompatibility^21^ and non-toxicity^22^, which is significant for applying body motion to trigger TENGs. Facile surface patterning and functionalization of PDMS provide the opportunity of controlling surface characterization of the TENG electrode^23–25^, which is a crucial feature to achieve active TENG-based sensors.

Among the environmental properties of the domestic and industrial atmosphere, detecting the amount of ambient humidity is significant^8^. Generally, the sensing material should be inclined to adsorb water molecules, due to its porosity and/or surface adsorbing sites, such as defects and chemical groups^26^. Since conventional polymers applied as TENG electrodes, like PDMS, are hydrophobic, they are not appropriate candidates for humidity sensing. On the other hand, the adsorption of water molecules on a nonporous surface is restricted up to the medium range of relative humidity (RH)^27^. However, these limitations can be modified by surface treatments, such as surface patterning, foaming, and compositing with hydrophilic additives^28^. Producing porosity generally can be performed via using dissolvable or evaporating materials to generate hollow spaces in the bulk polymer^29–31^. Among the nanomaterials, carbon nanotube (CNT) has been widely used as an additive, to obtain a nanocomposite foam with desirable sensing behavior and power generation properties^32–35^.

Here, we proposed a straightforward method to synthesize PDMS foam with water addition to produce macro- and micro-porosity. To reduce the hydrophobicity of PDMS, CNT as an aqueous solution was added to the polymer. During this method, the addition of nanomaterials in aqueous solutions is feasible, leading to a biocompatible surface, due to the fact that nanomaterials will be surrounded by PDMS. Applying the composite of PDMS with CNT as a TENG electrode resulted in enhancing humidity sensing response, as well as widening the sensing range of detecting RH. In addition to the novelty of foaming and compositing PDMS without toxic solvents, an uncomplicated humidity sensor was introduced through an active TENG-based setup, promising for the future of self-powered sensing networks.

## Results and discussion

### Characterizing the produced electrodes

**Figure 1:**
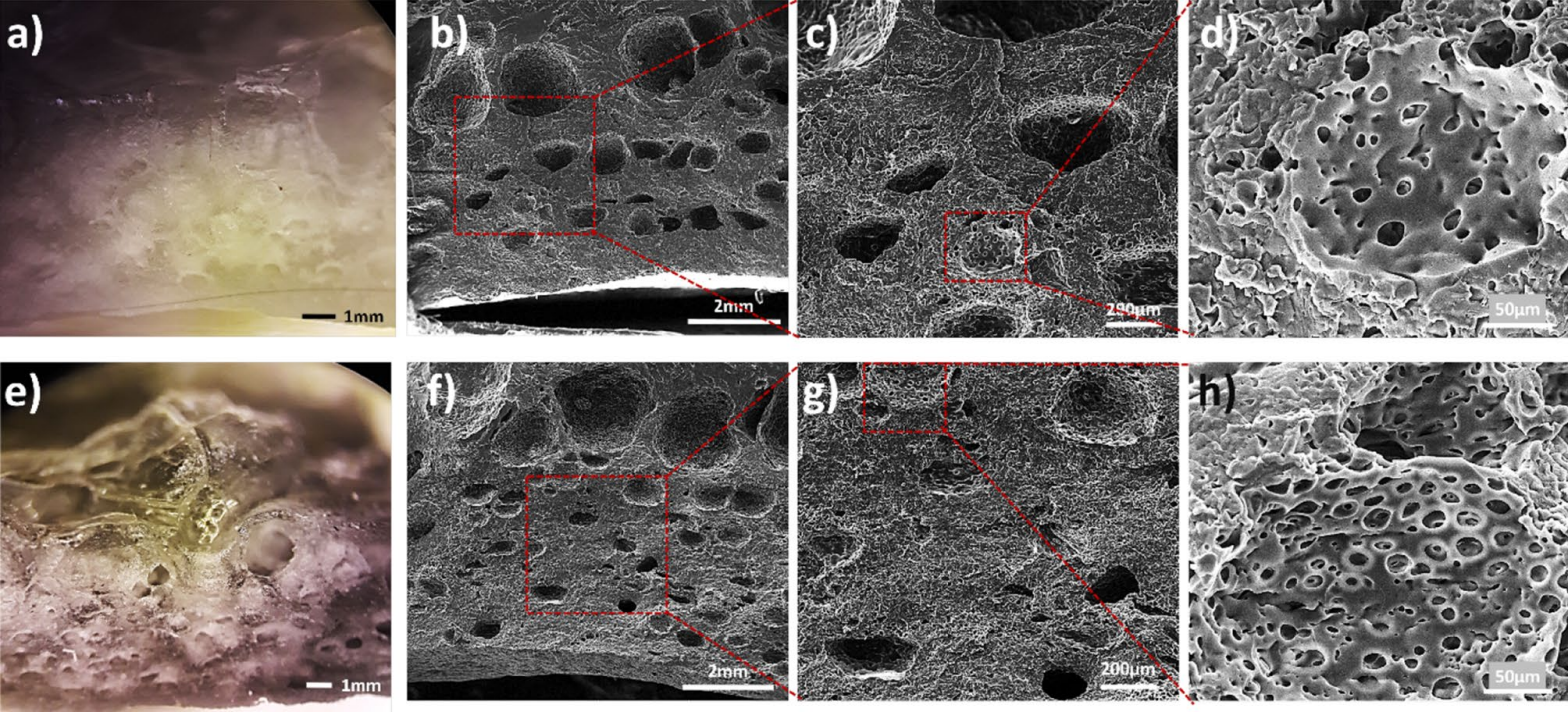
Optical (**a**, **e**) and SEM (**b**–**d**, **f**–**h**) images of the cross-section of pure porous PDMS (top row) and CNT-PDMS (bottom row) electrodes.

Figure 1 represents the porosity of PDMS and CNT-PDMS foams in various magnifications of the cross-section. The optical images of the polymer shown in Fig. 1a,e demonstrate the increase in the number of macroscopic pores by the addition of CNT. Since CNT is hydrophilic, it helps more efficient dispersion of the water drops within the polymer, resulting in higher value of porosity after the evaporation of trapped water. Figure 1b and f show the macro-pore distribution in the foam, where larger pores can be observed near the surface. Meanwhile, the trapped water is moving toward the surface, they are inclined to join together, due to the hydrophobicity of PDMS. In the presence of CNT, it is expected that the hydrophilicity of the matrix improves, and consequently, a smaller pore size is achieved. Figure 1c,d,g, and h demonstrate micro-pores within the macro-size pores, especially in CNT-PDMS nanocomposite.

**Figure 2:**
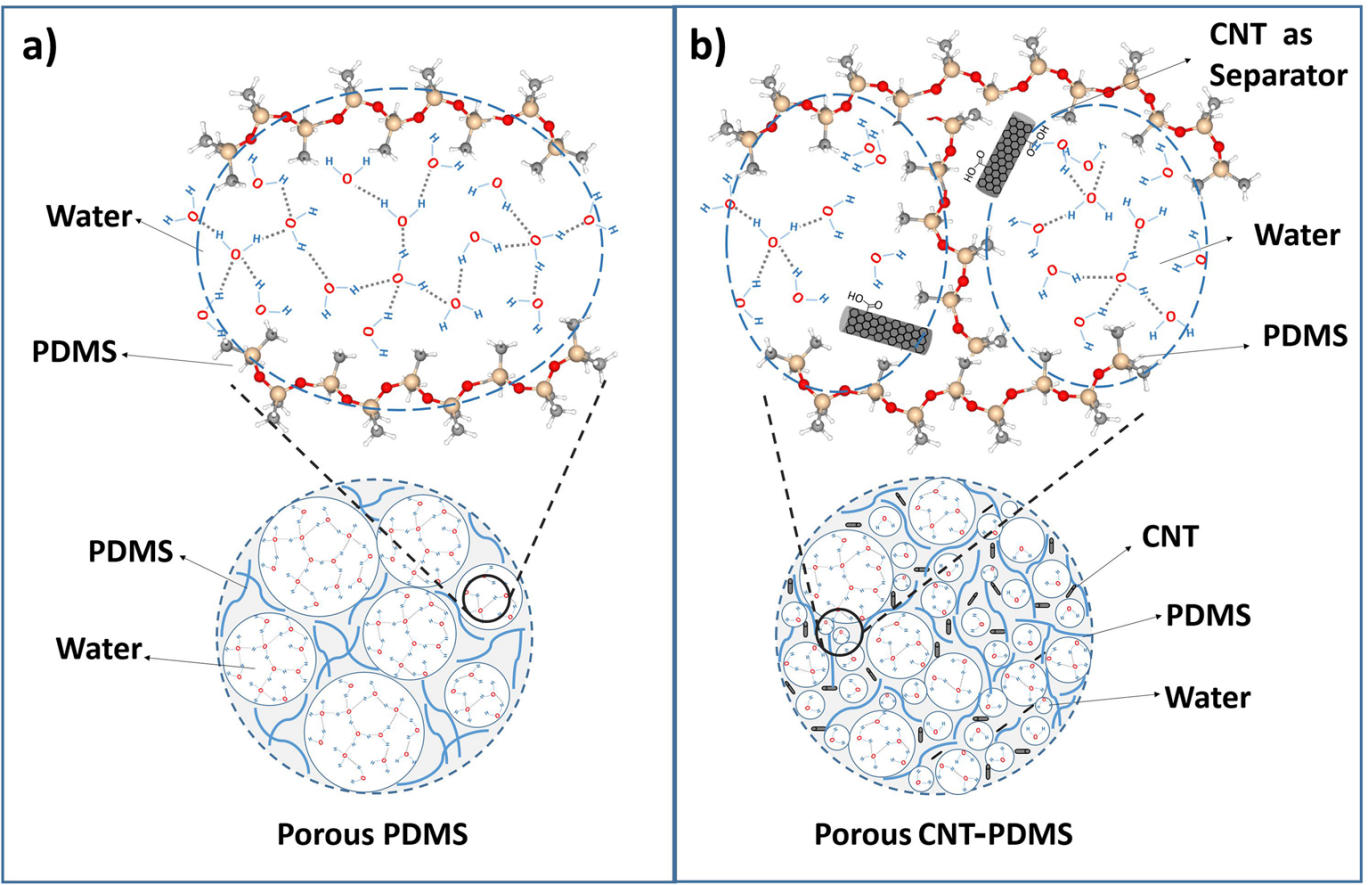
The formation of pores containing water molecules inside the PDMS network (**a**) and the CNT-PDMS network (**b**), where the presence of carbon nanotubes as a separating agent of water molecules causes the creation of smaller pores.

Figure 2 demonstrates the mechanism of water entrapment inside the polymer. By stirring the polymer precursors and water, the colonies of water produced inside the polymeric network (Fig. 2a). Then, heating up the whole system resulted in both polymer curing and water evaporation, leading to obtaining a porous structure. By addition of CNTs (Fig. 2b), the functional groups on the CNT (confirmed by FTIR analysis (Figure S1)) form several hydrogen bonding with water molecules, facilitating water entrapment inside the polymer network during stirring. Therefore, smaller amounts of water would be trapped inside the polymeric network in CNT-PDMS (Fig. 2b) compared to PDMS (Fig. 2a), resulting in finer pores observed in the final sample after the evaporation of water.

**Figure 3:**
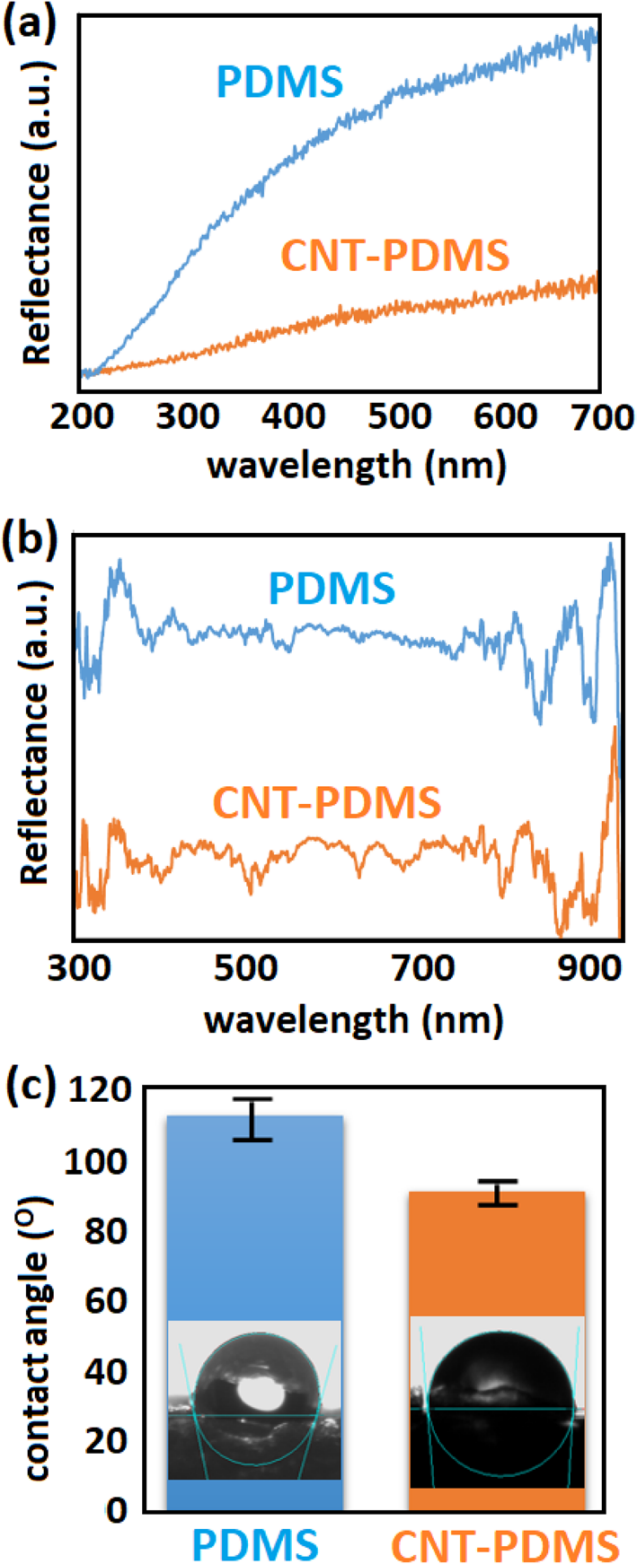
Characterization of fabricated foams. UV–Vis (**a**), DRS (**b**), and contact angle (**c**) tests.

The presence of CNT leads to an obviously darker color of the obtained polymer (Figure S2). Since the fabricated foams are opaque (in contrast to dense PDMS), their reflection behavior was investigated via UV–Vis spectroscopy (Fig. 3a). According to the absence of transmittance, the lower reflection of CNT-PDMS confirms higher absorption by CNTs. DRS spectroscopy was also performed, due to the highly porous surface of the electrodes, which reveals analogous result for CNT-PDMS compared to pure PDMS (Fig. 3b). On the other hand, the amount of carbon atoms is higher in specific points of the elemental analysis (Figure S3), confirming the presence of CNTs. EDX map also shows the distribution of pores within the whole thickness of the foam (Figure S4). The reduction of hydrophobicity of the PDMS after the addition of CNT was investigated via contact angle test, as shown in Fig. 3c. Accordingly, more inclination to water molecules is expected, resulting in finer porosity as mentioned above.

### TENG studies

**Figure 4:**
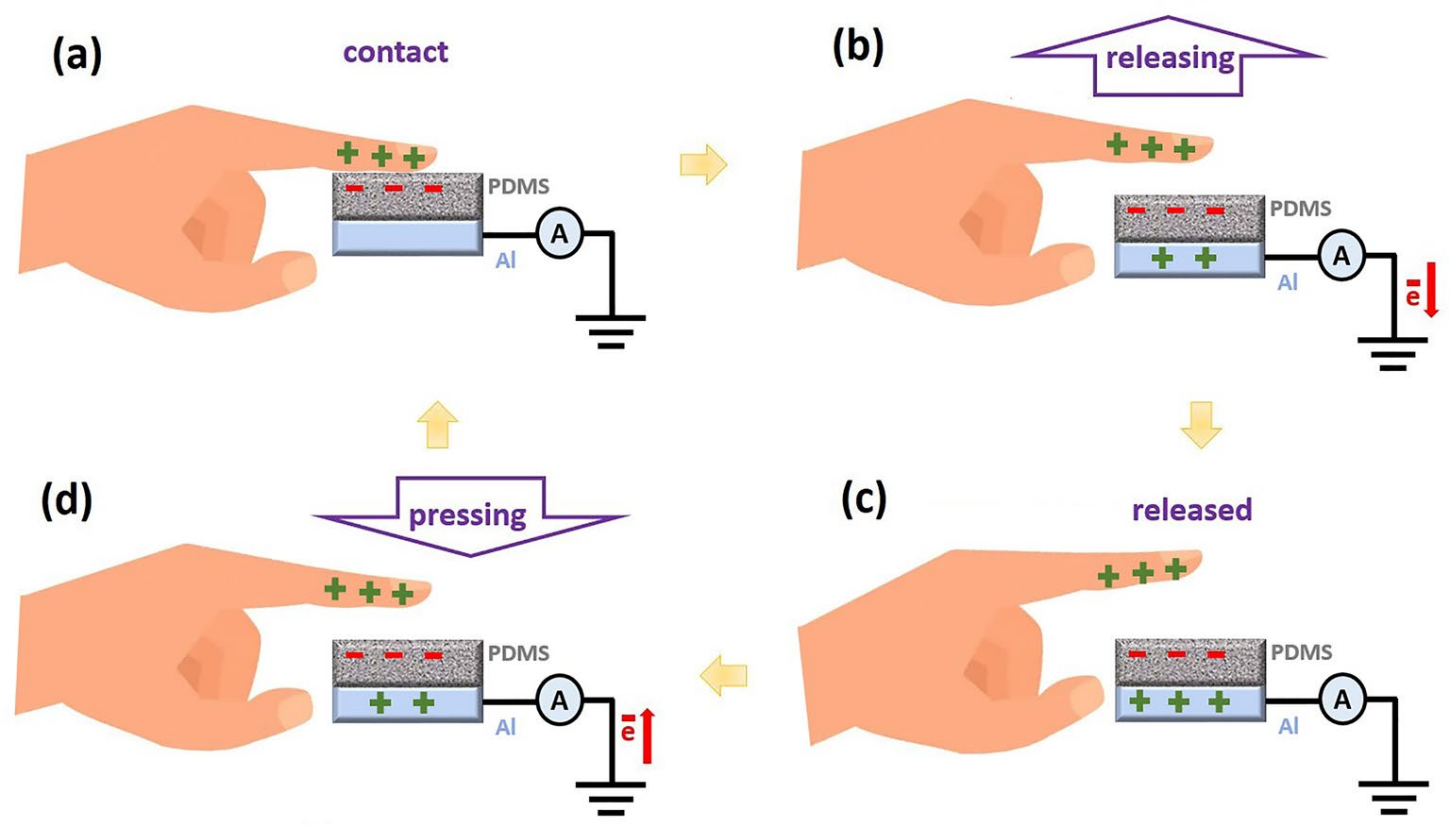
TENG mechanism. The generated charge in contact (**a**), releasing (**b**), released (**c**), and pressing (**d**) steps.

To investigate the TENG performance of the fabricated electrodes, the output voltage and current of three single-electrode TENGs were studied. According to the TENG structure shown in Fig. 4, the mechanism of power generation via finger tapping can be observed. When the finger is in contact with the PDMS electrode, negative triboelectric charges are produced on the surface of the polymer (Fig. 4a). During the releasing step, negative charges move from aluminum toward the ground, in order to satisfy the equilibrium condition (Fig. 4b). In the released step, the whole system is stable, resulting in no charge transfer (Fig. 4c). In the pressing step, the electrons move in the reverse direction (Fig. 4d), which leads to an inverted signal peak.

**Figure 5:**
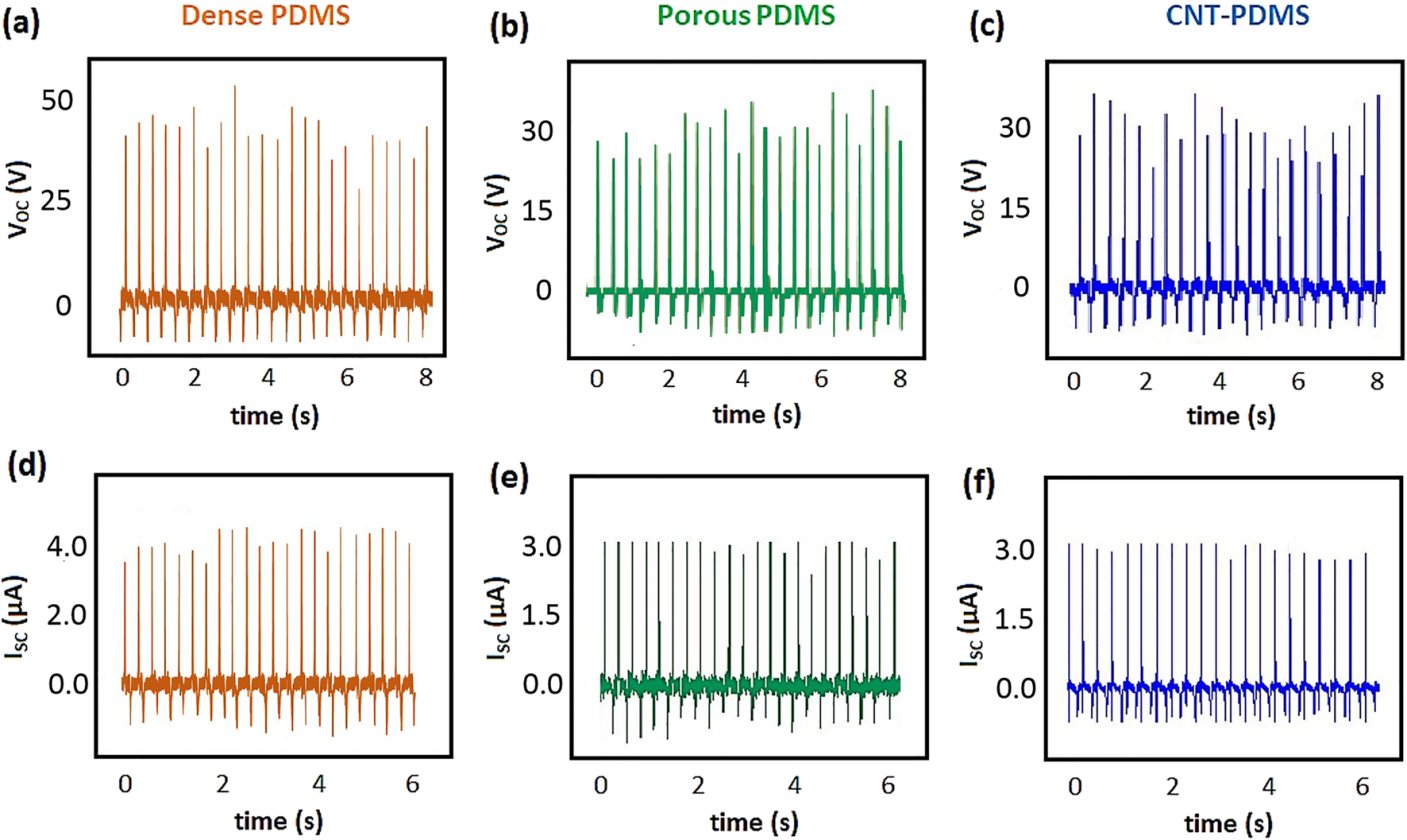
Generated voltage and current for TENGs based on dense PDMS (**a** and **d**), porous PDMS (**b** and **e**), and CNT-PDMS (**c** and **f**).

Figure 5 demonstrates the open-circuit voltage and short-circuit current of the TENGs at room temperature and humidity. The mean values of the output voltage of TENGs based on dense, porous and CNT-added PDMS were 35 ± 19 V, 30 ± 13 V, and 27 ± 15 V, respectively (Fig. 5a–c). Similarly, the average amount of output current is higher for dense PDMS (4.1 µA), compared to the porous electrodes generating peaks with maximum values of about 3 µA (Fig. 5d–f). Since the variations of maximum peaks of current were slighter than the voltage diagram, humidity sensing was performed by recording the output current.

The reduction of produced electricity via porous electrodes was resulted from a lower dielectric constant. According to Eq. 1, the induced charges on the back-contact, demonstrating the amount of short-circuit current, depending on the surface charge density (σ) and dielectric constant of the polymeric electrode (ɛ):^36^

1 $$ {\text{Q}}_{{{\text{SC}}}} = {\text{S}}_{{\upsigma {\text{x}}}} ({\text{t}})/\left( {{\text{d}}/\upvarepsilon + {\text{x}}\left( {\text{t}} \right)} \right) $$

where S and d are the effective surface and the thickness of the electrode and x(t) represents the distance between the bottom and up electrodes, which is varying during every tapping. Since CNTs do not exist on the surface of CNT-PDMS (Figure S5), σ is similar for all electrodes. Regarding the fact that other parameters had similar values for all TENGs, the only effective factors should be S and ɛ. Surface porosity led to decreasing S, while the main reason for ɛ reduction was resulted from pores produced inside the electrode. Since ɛ of the polymer is higher than air, the porosity led to decreasing the amount of ɛ for both foam electrodes. Therefore, the triboelectric charge produced on the surface of the polymer reduced after the foaming process. The additional finer pores, which had been observed in SEM images, caused a little more reduction of ɛ for CNT-PDMS TENG.

### Humidity tests

**Figure 6:**
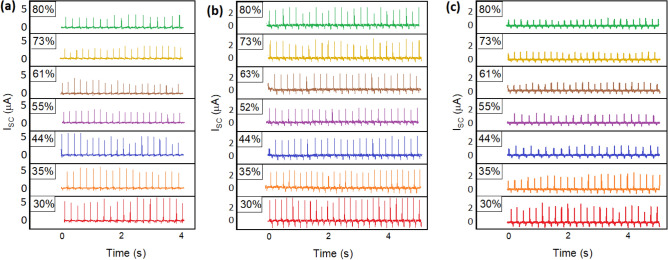
Variation of short-circuit current for TENGs based on dense PDMS (**a**), porous PDMS (**b**), and porous CNT-PDMS (**c**) electrodes.

**Figure 7:**
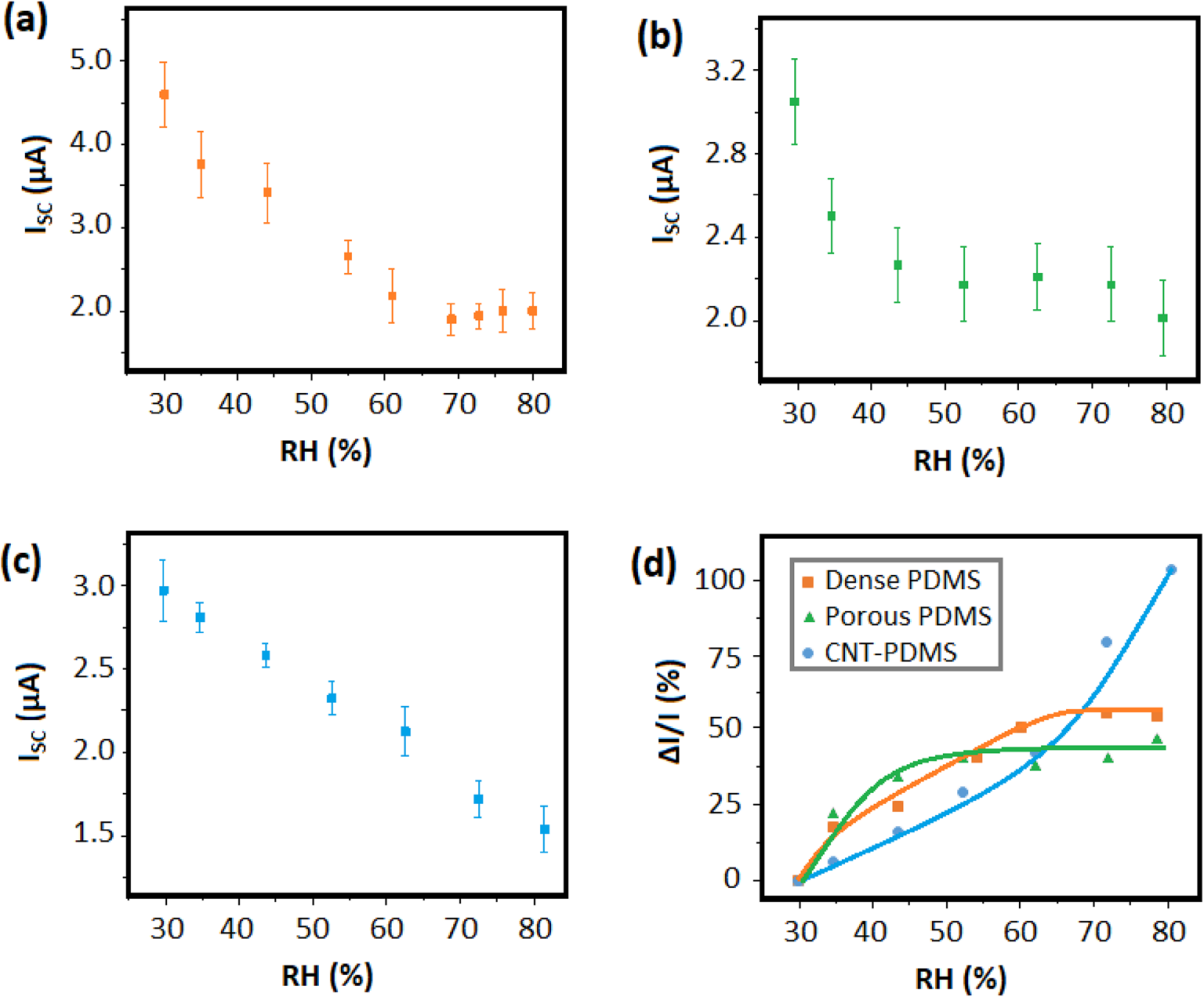
Sensing behavior of fabricated TENGs. Short-circuit current variations of dense PDMS (**a**), porous PDMS (**b**), and CNT-PDMS (**c**) based TENGs against RH values. Comparison of current response vs. RH for TENG-based humidity sensors (**d**).

Figure 6 demonstrates the variation of the output current of the fabricated TENGs under different values of RH. By increasing the ambient humidity, the surface charge density of electrodes diminished gradually. For dense and porous pure PDMS (Fig. 6a,b), the reduction of current does not proceed up to higher values of RH, while for CNT-PDMS, decreasing the output signal is observable within the whole range of RH (Fig. 6c). To demonstrate the variations of output current, the I_OC_-time diagrams were sketched as shown in Fig. 7a,b,c, which show the sensing behavior of the self-powered sensors as discussed above.

As shown in Fig. 7a–c, the reduction of output current shows diverse behaviors for the fabricated TENGs. By increasing the RH around the dense PDMS electrode, the generated current decreased gradually to reach a saturation value of 2 µA at RH > 60% (Fig. 7a). Therefore, no effective sensing is reported at higher amounts of RH. For porous PDMS, the saturation was observed at lower amounts of RH by the mean value of 2.2 µA (Fig. 7b). In contrast to pure polymer electrodes, CNT-PDMS was able to sense RH variation within the whole range of 30–80% (Fig. 7c). The output current gradually decreased from 2.9 to 1.6 µA by increasing RH from 30% up to 80%. Moreover, the linear behavior of humidity sensing facilitates the process of data analysis^37^. On the other hand, the precision of the sensor, which is defined as the dispersion of obtained data at the desired RH, differs apparently for each electrode. According to Fig. 7a–c, the error bars for gathered data are obviously lower for CNT-PDMS TENG, compared to the pure PDMS sensors.

According to Eq. 1, discussed in TENG Studies Section, the amount of charge induced on the back-contact electrode via finger tapping is proportional to the amount of surface charge density (σ). Since the amount of σ decreases by elevating the ambient humidity, smaller values of charge generate at higher RH^38^. Consequently, the transferred charges in the circuit declined for every tapping cycle, leading to lower amounts of short-circuit peaks in the diagrams shown in Fig. 6. This reduction either continues within the whole range of RH values or might be restricted up to a certain amount of RH, depending on the nature of the sensing electrode^27^.

To investigate the obtained data more thoroughly, the response values of current against the relative humidity were calculated employing the following formula: (I_0 _− I)/I. Here, I_0_ and I represent the quantities of current at RH = 30% and the desired RH, respectively. According to Fig. 7d, showing the curves of humidity response of the electrodes, the CNT-PDMS electrode represents the highest response to humidity variations, up to 108% for RH = 80%. This is about two times of the highest values for dense and porous PDMS, which were 56% and 50%, respectively. Therefore, the addition of CNT improved humidity response by more than 100%.

For both dense and porous PDMS, a saturation of humidity sensing was observed at higher amounts of RH. In contrast, for CNT-PDMS, the current decreased continuously up to high ambient humidity circumstances, due to the wide-range of pore distribution. For the primer electrodes, physisorption of water molecules reached a maximum value at a specific RH, and consequently, no more molecules had the opportunity of diffusing toward the surface of the electrode. For the CNT-PDMS electrode, alongside the saturation of adsorption in larger pores, the diffusion proceeds inside the smaller ones, which requires higher pressure of water molecules to initiate. This phenomenon occurred due to the hydrophilicity of CNTs, especially where presented on the edge of the pores, providing water diffusion into smaller pores. Therefore, the current is reduced by the physisorption of water molecules into a wide-range-size of pores.

**Figure 8:**
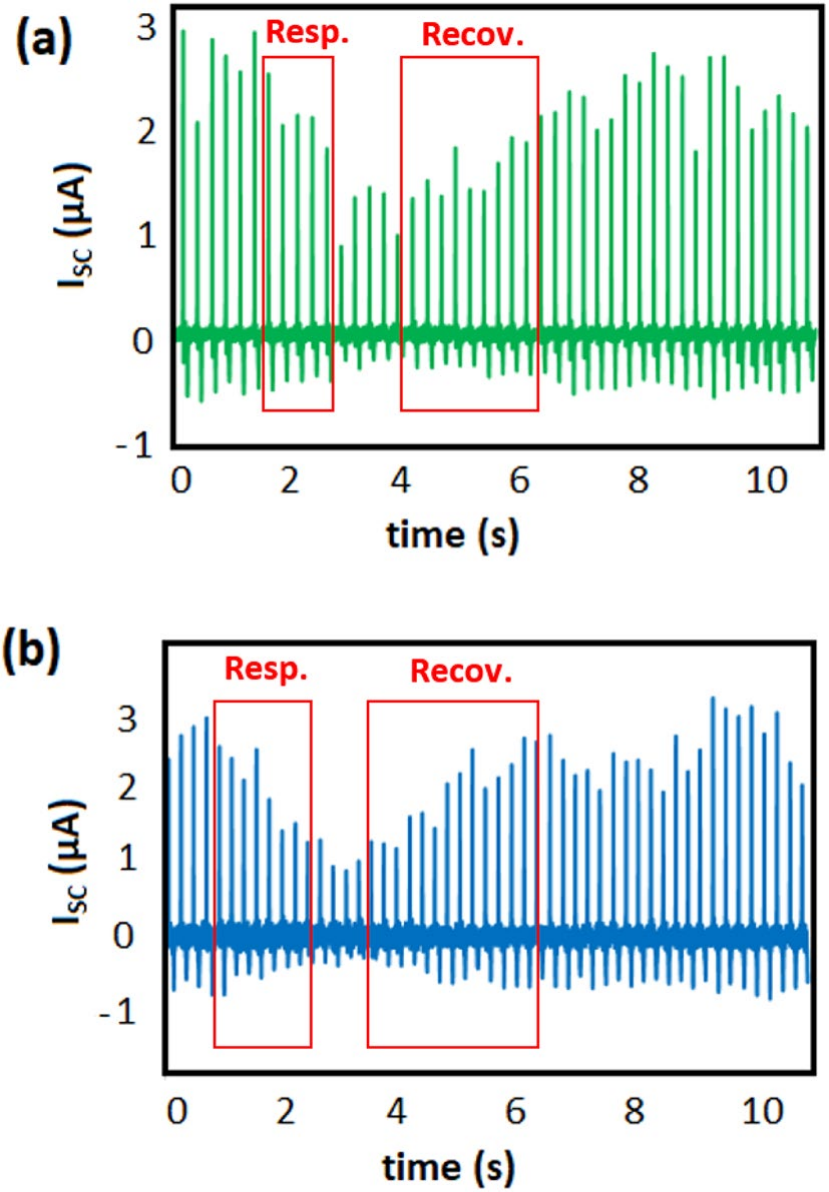
Dynamic response of porous PDMS (**a**) and CNT-PDMS (**b**). Recovery and response times of humidity sensing are indicated.

However, the above-mentioned mechanism led to delaying response and recovery sensing behavior for CNT-PDMS TENG compared to the porous pure polymer (Fig. 8). The response and recovery times are defined as the recorded time to reach 90% of the considered value. To measure the amounts of response and recovery times, we applied a setup consisting of two chambers with RH = 30% (the ambient humidity of the laboratory) and RH = 90% (provided by a humidifier) (Figure S6). The two chambers are connected via an aperture, by means of which the humidity can be augmented abruptly. For pure porous PDMS, the recovery time is 1.3 s, while it is 2.1 s for CNT-PDMS. Analogously, the recovery time after the addition of CNT increased from 1.8 s to 2.5 s. Therefore, adding CNT to PDMS results in the improvement of sensitivity, as well as precision, while simultaneously leading to a trivial decrease in the response and recovery rates, due to the finer porosity generated in the foaming process.

The sensing characteristics of the fabricated CNT-PDMS sensor are shown in Table 1 compared to the previous studies. The first outstanding feature of current work is not only being self-powered, due to applying TENGs but also the integration of the whole setup, resulting from the active circuit design. Moreover, the fabricated foams are highly light and flexible, beneficial for wearable/portable applications. Compared to previous investigations based on PDMS, CNT, or TENG-based polymeric sensors, the response and recovery times of our humidity sensor are appreciable. The proposed foaming procedure in this research opens a new avenue of facile nanocomposite production, appropriate for enhancement of gas sensing.

**Table 1:** Comparison of humidity sensing characteristics of similar previous investigations with our fabricated CNT-PDMS sensors.

Type	Sensing material	Flexible/Rigid	Response time (s)	Recovery time (s)	Ref
Capacitive	nanofibrillated cellulose/graphene oxide/PDMS	Flexible	57	2	^39^
Impedance	Armalcolite/PDMS	Flexible	10	15	^40^
Resistive	CNT	Rigid	40	60	^41^
Self-powered	PFSA*	Flexible	30	–	^42^
Passive TENG	PTFE	Rigid	–	–	^43^
Passive TENG	rGO/PVP	Rigid	2.8	3.5	^44^
Active TENG	CNT-PDMS	Flexible	1.8	2.5	This work

*perfluorosulfonic acid ionomer.

## Conclusions

In summary, our proposed method for facile fabrication of PDMS-based foams was investigated by the addition of CNT. Sensing features of the obtained electrode were totally outstanding, as follows: Response values of 108% (improved by more than 100%); RH range broadens to higher values of 80%; the response/recovery times remained at about 2 s. These mentioned enhancements were achieved by applying a straightforward method of simultaneous addition of porosity and hydrophilic fillers. The proposed self-powered humidity sensor based on PDMS is extremely promising for portable electronics, as well as industrial applications.

## Experimental section

### Preparation of the electrodes

**Figure 9:**
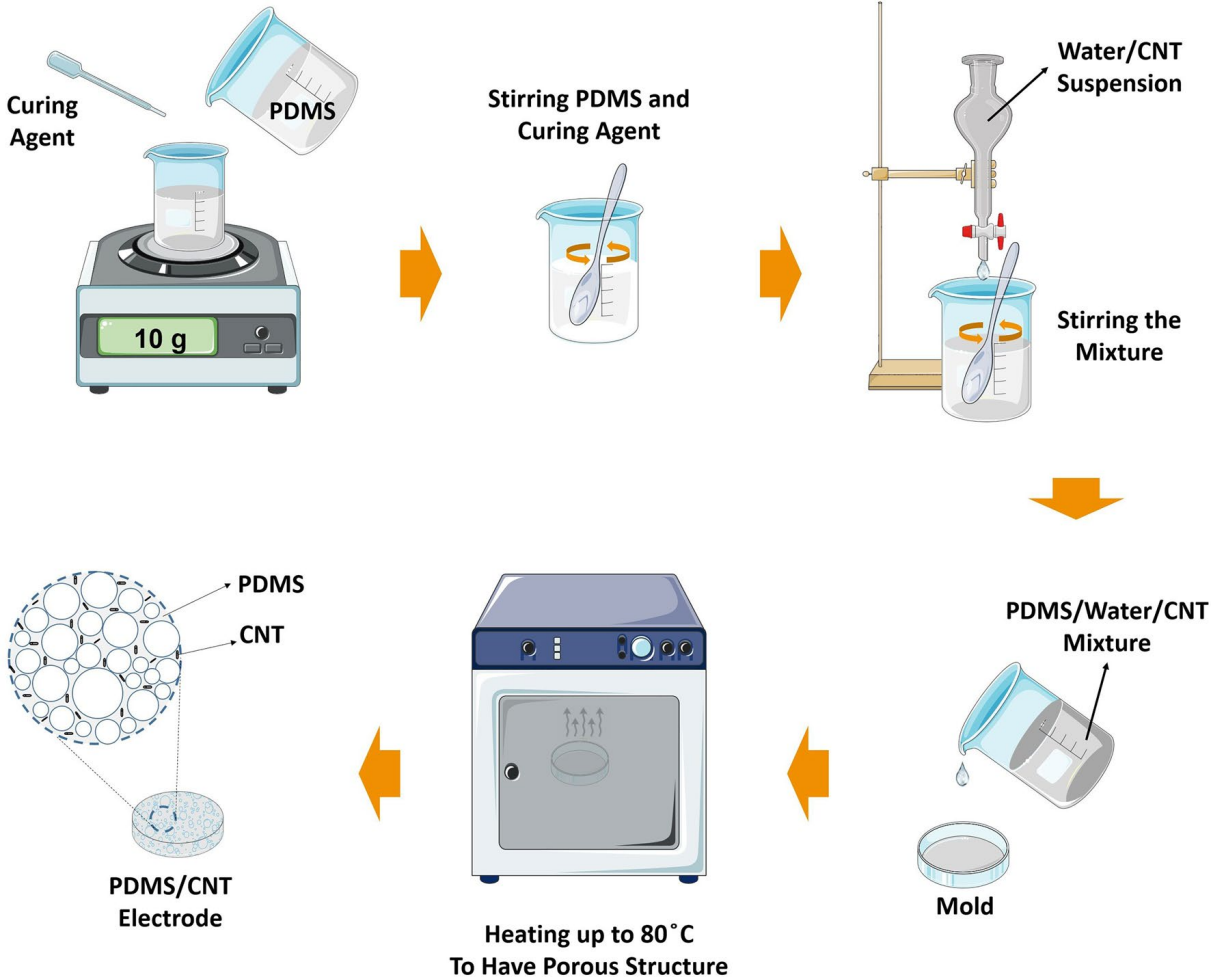
Schematic of the fabrication steps of PDMS/CNT porous electrode.

In order to fabricate the electrodes, Sylgard 184 PDMS elastomer was mixed with a curing agent at a weight ratio of 10:1 and stirred well enough. Functional groups were produced on the wall of CNT by refluxing it with H_2_SO_4_/HNO_3_ (3:1 v/v), as described in the literature^45^. Then the suspension composed of a mixture of water and CNT (4 mg/ml) was added drop by drop to the polymer mixture. To ensure uniform dispersion of nanotubes in water, the suspension was placed in an ultrasonic bath for 15 min before use. During the addition of the suspension of water and CNT, stirring was continued until finally a greyish-white mixture was obtained. It should be mentioned that due to the presence of trapped water in the polymer network, the volume of the resulting mixture increased to about twofold of the initial volume. Then the resulting mixture was poured into the desired mold. Thereafter, the sample was placed in an oven with a temperature of 80 °C for 2 h to evaporate the water trapped in the polymer networks, as well as to cure the polymer. Finally, the sample was slowly removed from the mold. This electrode manufacturing method is straightforward and inexpensive, without the requirement of special/toxic chemicals and complex devices as well. Figure 9 demonstrates the schematic of the fabrication steps of the PDMS/CNT porous electrode.

### TENG fabrication

The power generation was achieved by finger tapping on the prepared electrodes in single-electrode mode. PDMS-based electrodes were placed as the bottom tribo-material, while the human skin was accounted for as the top electrode of the TENG. The output signals were recorded via the aluminum foils, which had been attached to the bottom of the polymers. The other probe of the recording devices had been grounded. Tapping was performed by two fingers, hence, the active area participating in triboelectric power generation had no alteration.

### Humidity tests

The electrode was placed inside a flexible chamber, which let the user’s hand comfortably enter the chamber to perform tapping. A commercial hygrometer was located close to the electrode to record the RH. The ambient humidity was regulated by means of a conventional humidifier, whose outlet pipe was fixed in an aperture created on the wall of the chamber. The output signals were transferred via a coated wire passing through the wall of the surrounding chamber.

### Instruments and characterization

Optical and SEM images were captured by Stereo J5 and Tescan (MIRA3-15 kV) electron microscope, respectively. UV–Vis spectra, as well as DRS spectroscopy, were performed via a Lambda25 (Perkin–Elmer, USA) spectrophotometer. FTIR spectroscopy was carried out by means of a Spectrum RX I (Perkin–Elmer, USA) to detect chemical groups on the wall surface of CNT. An oscilloscope (DSO1022A) and an Ivium Compactstat were applied to record the generated voltage and current, respectively. The contact angle tests were carried out under the 3006-0102-7 standard condition.

## Supplementary Information

Supplementary Video 1.

Supplementary Information 1.

## Data Availability

The datasets used and/or analyzed during the current study available from the corresponding author on reasonable request.
