# Supplementary material for: CNT-PDMS foams as self-powered humidity sensors based on triboelectric nanogenerators driven by finger tapping
Source: Sci Rep. 2023 Jan 7;13:370. doi: 10.1038/s41598-023-27690-5 (PMC9825370; doi:10.1038/s41598-023-27690-5)
Supplement: Supplementary file 2 — Supplementary Information 1. [file 41598_2023_27690_MOESM2_ESM.docx]

**CNT-PDMS foams as self-powered humidity sensors based on triboelectric nanogenerators driven by finger tapping**

Mohaddeseh Vafaiee, Faezeh Ejehi, Raheleh Mohammadpour^*^

Institute for Nanoscience and Nanotechnology, Sharif University of Technology, Tehran 14588-89694, Iran

*E-mail: mohammadpour@sharif.edu


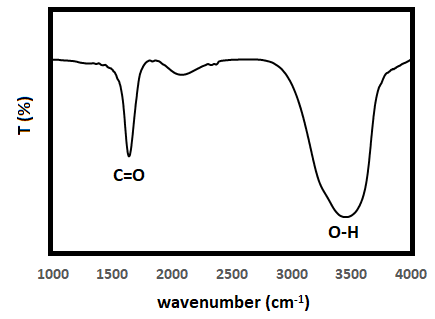


**Figure S1.** FTIR spectrum of carbon nanotubes following acid treatment. The main peaks at 3459 and 1638 cm^-1^ correspond to O-H and C=O bonds of carboxyl groups, respectively.


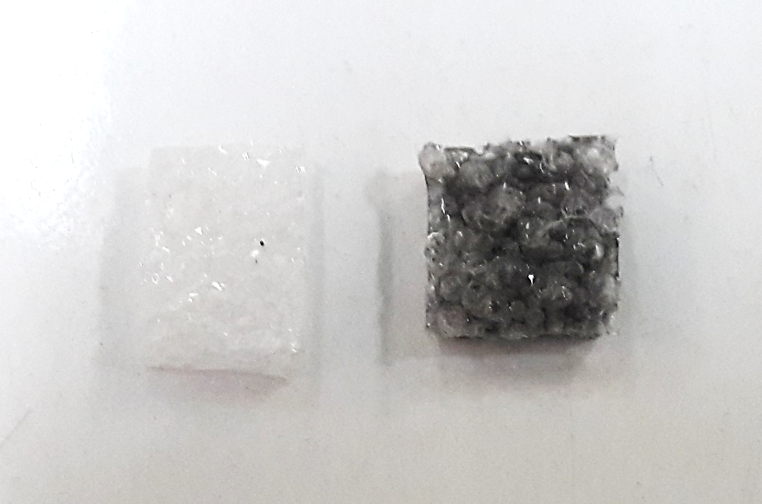


**Figure S2.** Optical image of the prepared foams: PDMS (left) and CNT-PDMS (right).


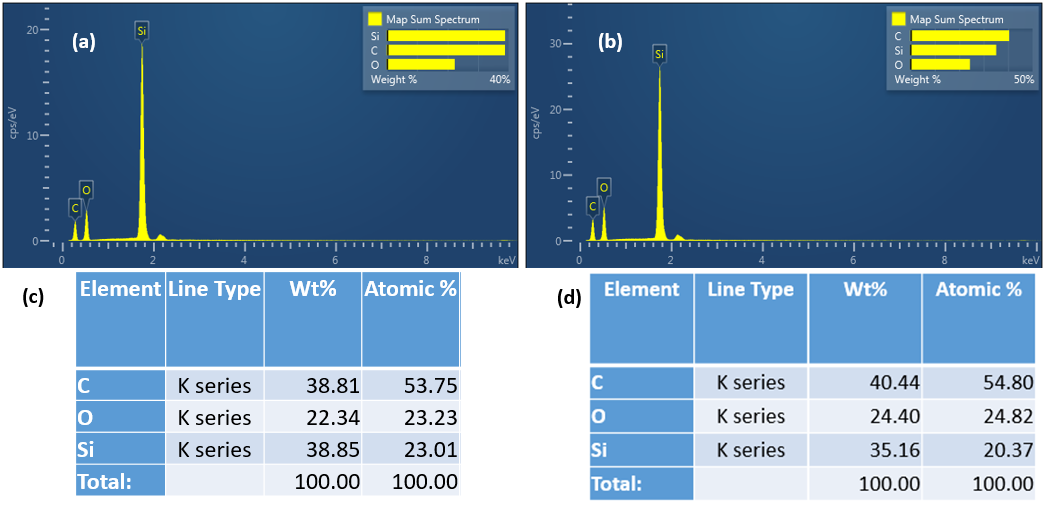


**Figure S3.** EDX Elemental analysis of the fabricated foams: percentage of carbon, oxygen, and silicon atoms for porous PDMS (a,c) and CNT-PDMS (b,d).


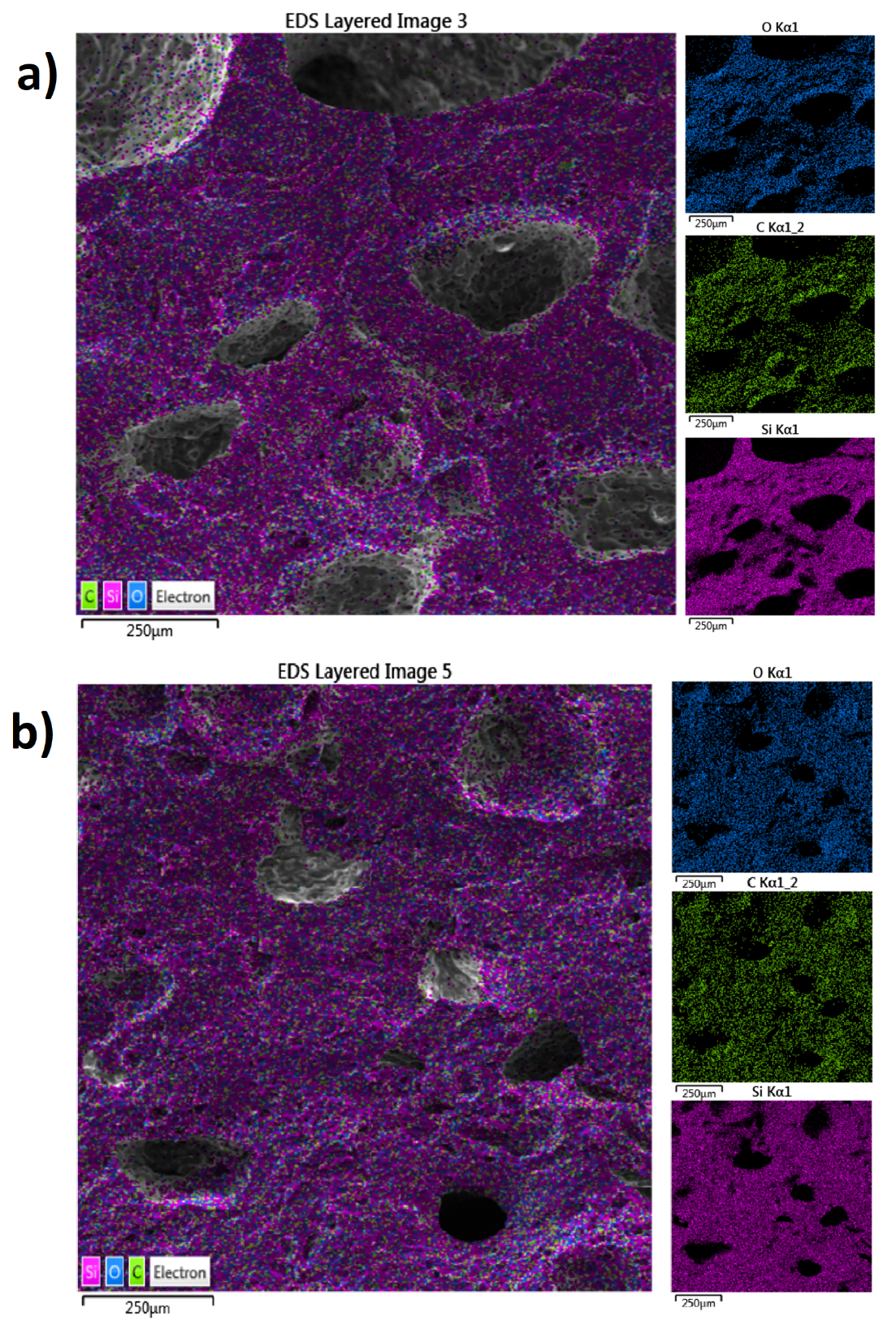


**Figure S4.**  SEM and EDX/Map analysis of porous PDMS (a) and CNT-PDMS (b).


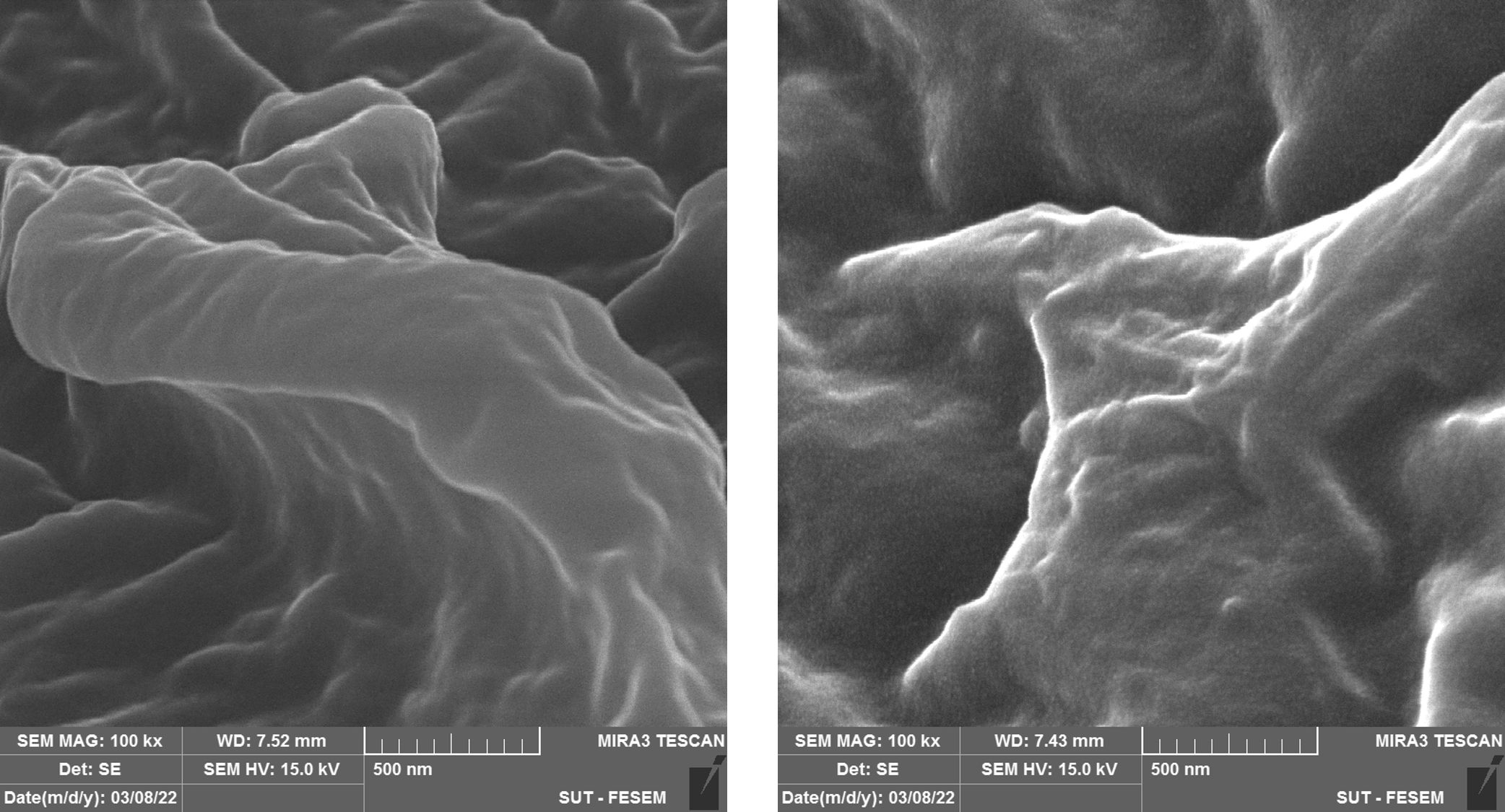


**Figure S5.** No CNT was observed in SEM images of the surface of PDMS-CNT foam.


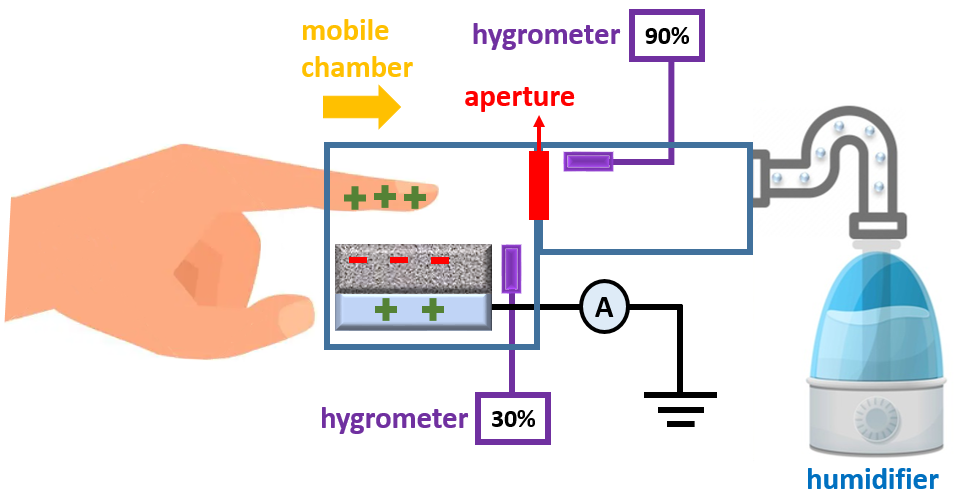


**Figure S6.** Response/recovery time measurements for PDMS-based TENG. The high humidity atmosphere was achieved in the right chamber, while the ambient RH of the left one is 30%. The mentioned aperture should be opened for calculating the response time. After 1 second, the aperture was removed, and consequently, the electrode was exposed to the air (RH = 30%).


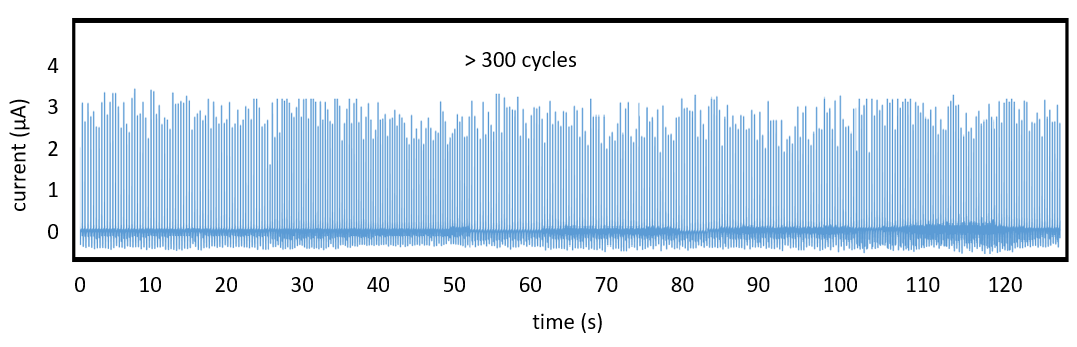

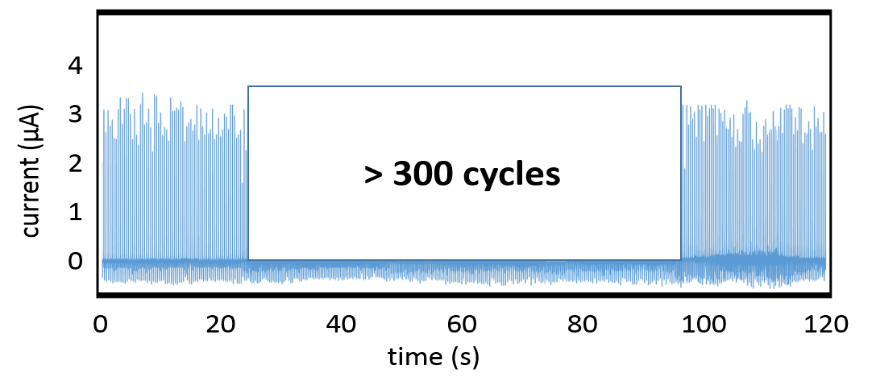

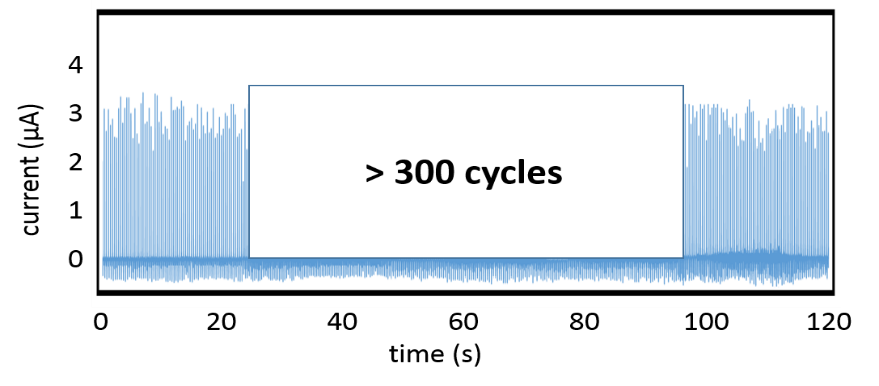


**Figure S7.** Longtime stability of PDMS-CNT TENG triggered via finger tapping.

**Caption for Supplementary Movie S1:**

Real-time voltage generation by finger tapping on the CNT-PDMS electrode. The output peaks were revealed on the screen of the oscilloscope and vanished as soon as tapping stopped. One of the oscilloscope terminals was connected to the back-contact electrode (i.e. aluminum foil), while the other one was grounded.
